# A Study of the Clinical Effects of Sequential Combined Spinal Epidural Anesthesia and Spinal Anesthesia in Patients Undergoing Orthopedic Surgeries

**DOI:** 10.7759/cureus.39171

**Published:** 2023-05-18

**Authors:** Mahima LN, Ravi Madhusudhana

**Affiliations:** 1 Anaesthesia, Sri Devaraj URS Medical College, Kolar, IND

**Keywords:** epidural anaesthesia, orthopedic surgeries, analgesia, spinal anaesthesia, sequential combined spinal epidural anaesthesia

## Abstract

Background: In orthopedic surgeries, there has been an increase in the usage of the neuraxial blockade to provide excellent surgical conditions and prolonged postoperative analgesia. The introduction of the sequential combined spinal epidural anesthesia (SCSEA) technique provides benefits for both spinal anesthesia (SA) and epidural anesthesia. The focus of this study was to analyze the time needed to attain a desired level of sensory block, to compare the period of sensory block, and to study the intraoperative hemodynamics in the SCSEA and SA groups.

Materials and methods: The study was conducted on patients admitted for elective lower limb orthopedic surgeries. The sample size for this prospective randomized study is two groups of 67 subjects each. Patients aged between 18 and 65 years, posted for orthopedic surgeries for two to three hours, and of American Society of Anaesthesiologists (ASA) Grades 1 and 2 were included and divided into two groups. Group A patients received SCSEA with an epidural-test dose of 3 ml lignocaine (2%) with adrenaline and spinal bupivacaine (0.5%) of 1.5 ml-7.5 mg + fentanyl .25 mic if the sensory level was below T8. An epidural top-up was given with 2 ml per segment of 0.5% bupivacaine to bring the sensory level to T8. Group B patients received SA with spinal bupivacaine (0.5%) of 3 ml-15 mg + fentanyl .25 mic. Intraoperative hemodynamics, the duration to achieve a sensory level of T8, the time for two-segment regression of sensory block, and the complications that occurred were recorded.

Results: The study included a total of 134 subjects with each group having 67 subjects admitted for lower limb surgery. The mean value (SD) of the time taken to attain sensory block in the SCSEA group was prolonged when compared to the SA group (7.15 ± 0.75 and 5.01 ± 0.88). The time for two-segment regression in the SCSEA group was 86.77 ± 3.60 and the SA group was 106.4 ± 8.01, which indicated that the SA group has a longer and better sensory block. Substantially, the study shows that the SCSEA group (P < 0.05) has better hemodynamics when compared to the SA group.

Conclusion: The SCSEA technique has better intraoperative hemodynamic stability with a longer analgesic effect when compared to SA. SA shows a sudden change in hemodynamics but reveals a greater sensory block.

## Introduction

Spinal anesthesia (SA) was the first regional anesthetic procedure used, and it was performed by August Bier in 1898 in Germany [1]. The efficacy of SA in orthopedic surgery is in contrast to that of general anesthesia [2]. SA is an effective procedure as it has a high success rate of 90% [3]. On the other hand, epidural anesthesia allows for continuous but intermittent delivery of analgesic and anesthetic agents intraoperatively and postoperatively, allowing optimal treatment for intraoperative and postoperative pain in orthopedic surgeries [4].

SA has been observed to exhibit adverse effects such as hypotension, bradycardia, post-dural puncture headache, decreased body temperature, and a shorter duration of action [5,6]. Epidural anesthesia necessitates a large volume of local anesthesia with a greater concentration and a later onset. In epidural anesthesia, hypotension and bradycardia occur at a slower rate, providing adequate time for addressing hemodynamic alterations. Sequential combined spinal epidural anesthesia (SCSEA) offers rapid onset, longer duration of action, efficacy, and minimal toxicity and has various benefits such as stable hemodynamic status, better control of the duration of anesthesia, and delivery of postoperative analgesia [7].

Hence, to overcome the disadvantages of SA, SCSEA is used for most orthopedic surgeries. When Soresi introduced SCSEA in 1937, it used a single needle-single interspace approach [8]. With time, SCSEA has been proven beneficial, yielding more stable hemodynamics, longer blocking, and postoperative analgesia [9].

## Materials and methods

This study was conducted on patients admitted for elective lower limb orthopedic surgeries done at R. L. Jalappa Hospital and Research Centre, Sri Devaraj Urs Medical College, Tamaka, Kolar. Ethical approval was obtained from the Institutional Ethics Committee at Sri Devaraj Urs Medical College (SDUMC/KLR/IEC/128/2019-20)(SDUMC/KLR/IEC/128/2019-20). It was a randomized, prospective, comparative study, with a total sample size of 134 divided into two groups of 67 subjects each. The study period was from January 2020 to May 2021. Individuals aged over 18 years and below 65, of both male and female genders, and American Society of Anaesthesiologists (ASA) Grades 1 and 2 and patients posted for lower limb orthopedic surgeries two to three hours under subarachnoid block were included. On the other hand, the ASA Grades 3 and 4 patients, those with a bleeding disorder, on anticoagulant therapy, with local infection at the site of block, and with neurological deficits were excluded from the study.

Sampling procedure

Preoperatively, each patient was explained the procedure, and written informed consent was acquired from them. Patients were premedicated with alprazolam 0.5 mg at 10 PM before the day of the procedure and at 6 AM on the day of the procedure. The patients were divided into two groups: Group A and Group B (based on simple computer-generated randomization). For patients in the preoperative room, basal vitals were noted. The IV line (18G) was secured and preloaded with 500 ml of RL before the anesthetic procedure.

Group A patients received SCSEA. For its administration, an epidural catheter (20G) was secured at L2-L3 space using an 18G Tuohy needle, and the catheter was fixed after administering a test dose of 3 ml of lignocaine 2% with adrenaline (0.005 mg). The test dose was given to exclude the intravascular placement of an epidural catheter. Following this, SAB was performed at L3-L4 space using a 25G Quincke Babcock needle, and 1.5 ml (7.5 mg) of 0.5% hyperbaric bupivacaine with 25 mcg fentanyl was administered. The final sensory level achieved by the subject was noted, and if it was below T8, epidural top-up was given with 2 ml per segment of 0.5% bupivacaine to bring the sensory level to T8.

Group B patients received SA. SA was administered in a sitting position at the L3-L4 space using a 25G Quinke Babcock needle and 3 ml of 0.5% hyperbaric bupivacaine with 25 mcg of fentanyl. Patients were supine and the level of sensory block was monitored. Once the block level reached T8, the table was tilted to prevent further ascent of the sensory level.

The following data were recorded from both Group A and Group B subjects: time taken to achieve a sensory level of T8, the total dose of epidural bupivacaine required to establish the desired level of block, time for two-segment regression of sensory block, intraoperative hemodynamic parameters (heart rate and mean arterial blood pressure), supplementation with general anesthesia, and complications if any.

Hemodynamic variables, such as blood pressure (systolic, diastolic, and mean arterial blood pressure) and heart rate, were recorded before administering anesthesia and throughout the intraoperative period: a five-minute interval for the initial half an hour followed by every 10 mins afterward. If systolic blood pressure went below 90 mmHg or a 25% decrease in systolic blood pressure from baseline, 3-6 mg of mephentermine was administered intravenously. Bradycardia (heart rate <60 bpm) was treated with 0.6 mg atropine IV. In the postoperative period, patients in Group A received bupivacaine 0.125% 10 ml with 25 mcg fentanyl through an epidural catheter, and those in Group B received IV tramadol 50 mg on demand for pain relief. The patients were monitored for pain in the postoperative period through their visual analogue scale scores.

Statistical analysis

The sample size was calculated based on a study by Patel et al. [7]. The data were entered in Microsoft Excel and Microsoft Word (Microsoft, Washington, USA) and analyzed using SPSS Statistics version 22 (IBM Corp. Released 2013. IBM SPSS Statistics for Windows, Version 22.0. Armonk, NY: IBM Corp.). Qualitative data were presented in the form of proportions, whereas bar charts were used in graphical representation. Quantitative data were presented as mean and standard deviation. ANOVA was carried out to determine whether there were any statistically significant differences between the means of three or more independent (unrelated) groups. A p-value of <0.05 was considered statistically significant.

## Results

No significant difference in mean age groups was observed (Table 1). It was observed that 79.9% belong to ASA Grade 1 and 20% belong to ASA Grade 2.

**Table 1:** Age in years frequency distribution of patients in two groups

Age in Years	Group A (N=67)	Group B (N=67)	Total (N=134)
<30	22 (32.8%)	22 (32.8%)	44 (32.8%)
30-40	18 (26.9%)	22 (32.8%)	40 (29.9%)
41-50	12 (17.9%)	8 (11.9%)	20 (14.9%)
51-60	9 (13.4%)	9 (13.4%)	18 (13.4%)
>60	6 (9%)	6 (9%)	12 (9%)
Total	67 (100%)	67 (100%)	134 (100%)
Mean ± SD	39.11 ± 13.91	38.13 ± 14.86	38.62 ±14.35

No significant difference was noted in ASA grading (p-value = .518; Table 2).

**Table 2:** ASA grade frequency distribution of patients in the two groups

ASA Grade	Group A (N=67) (%)	Group B (N=67) (%)	Total (N=134) (%)
I	55 (82.1%)	52 (77.6%)	107 (79.9%)
II	12 (17.9%)	15 (22.4%)	27 (20.1%)
Total	67 (100%)	67 (100%)	134 (100%)

The time taken for the sensory blockade to commence was 7.15 ± 0.75 in Group A and 5.01 ± 0.88 in Group B. Compared to Group A, Group B exhibited a faster onset (Table 3). The time taken to achieve motor block in Group A was 9.64 ± 1 and in Group B it was 7.13 ± 0.8. Group B showed better motor blocking, and the time for two-segment regression in Group A was 108.34 ± 29.5, while it was 135.24 ± 12.88 in Group B. This indicates that Group B had a better sensory block (Table 3). There was a statistically significant difference between the two groups in parameters like anesthesia readiness time (minutes), the onset of sensory block (minutes), the onset of motor block (minutes), time to achieve T8 level (minutes), time for two-segment regression (minutes), duration of motor block (minutes), time for the first analgesic request (hours), and total bupivacaine consumption (milligram) (p-value <0.001). There was no statistically significant difference between the two groups in the duration of surgery (minute) (p-value 0.410).

**Table 3:** Comparison of study variables in the two groups

Variables	Group A (N=67) Mean ± SD	Group B (N=67) Mean ± SD	Total (N=134) Mean ± SD	p-value
Anesthesia readiness time (minutes)	13.19 ± 1.68	9.17 ± 0.96	11.18 ± 2.43	<0.001**
Onset of sensory block (minutes)	7.15 ± 0.75	5.01 ± 0.88	6.08± 1.35	<0.001**
Onset of motor block (minutes)	9.64 ± 1	7.13 ± 0.8	8.39 ± 1.55	<0.001**
Time to achieve T8 level (minutes)	12.92 ± 1.83	9.22 ± 1.24	11.07 ± 2.42	<0.001**
Duration of surgery (minutes)	105.82 ± 32.71	101.04 ± 34.21	103.43 ± 33.43	0.410
Time for two-segment regression (minutes)	108.34 ± 29.5	135.24 ± 12.88	121.79 ± 26.39	<0.001**
Duration of motor block (minutes)	167.39 ± 9.31	194.33 ± 14.35	180.86 ± 18.11	<0.001**
Time for the first analgesic request (hours)	7.01 ± 0.99	4.33 ± 0.87	5.67 ± 1.64	<0.001**
Total bupivacaine consumption (milligram)	40.3 ± 5.29	15 ± 0	27.65 ± 13.23	<0.001**

The baseline pulse rate was comparable in the two groups, which were 84.28 ± 10.38 and 84.48 ± 9.78 in Group A and Group B, respectively. There was a statistically significant difference between the two groups in pulse rate at five minutes, 10 minutes, and 50 minutes (p-value <0.05). There was no statistically significant difference between the two groups in the pulse rate at 0 minutes, 20 minutes, 30 minutes, and 40 minutes (p-value >0.05) (Table 4).

**Table 4:** Pulse rate comparison between the two groups

Pulse Rate (bpm)		Group A (N=67) Mean ± SD	Group B (N=67) Mean ± SD	Total (N=134) Mean ± SD	p-value
Baseline		84.28 ± 10.38	84.48 ± 9.78	84.38 ± 10.05	0.911
0 minutes		83.13 ± 9.94	83.82 ± 9.88	83.48±9.88	0.689
5 minutes		80.1 ± 9.36	74.61 ± 10.22	77.36 ± 10.14	<0.001**
10 minutes		78.01 ± 9.85	70.42 ± 13.48	73.22 ± 12.09	0.006**
15 minutes		74.54 ± 11.18	68.21 ± 10.22	70.87 ± 10.69	0.374
20 minutes		74.9 ± 11.19	70.43 ± 11.6	71.66 ± 11.38	0.336
30 minutes		75.51 ± 10.82	72.61 ± 11.74	74.56 ± 11.3	0.183
40 minutes		74.82 ± 10.99	78.81 ± 13	76.81 ± 12.16	0.058+
50 minutes		74.7 ± 12.09	79.64 ± 12.96	77.17 ± 12.73	0.024+
60 minutes		74.7 ± 12.33	80.61 ± 12.41	77.66 ± 12.68	0.007**
70 minutes		78.27 ± 16.12	82.64 ± 16.09	80.54 ± 16.19	0.126
70 minutes		99.17 ± 0.76	99.13 ± 0.74	99.15 ± 0.74	0.803

The baseline systolic blood pressure was 124.9 ± 13.02 and 116.61 ± 10.98 in Group A and Group B, respectively. There was a statistically significant difference between the two groups in baseline systolic blood pressure at 0 minutes, 5 minutes, 10 minutes, 15 minutes, 20 minutes, 30 minutes, and 40 minutes (p-value <0.05) (Table 5).

**Table 5:** Systolic blood pressure comparison of the two groups

Systolic Blood Pressure (mmHg)	Group A (N=67) Mean ± SD	Group B (N=67) Mean ± SD	Total (N=134) Mean ± SD	p-value
Baseline	124.9 ± 13.02	116.61 ± 10.98	120.75 ± 12.7	<0.001**
0 minutes	130.42 ± 14.57	126 ± 10.64	128.21 ± 12.9	0.047*
5 minutes	125.12 ± 12.42	111.88 ± 10.27	118.5 ± 13.15	<0.001**
10 minutes	120.36 ± 13.71	104.91 ± 10.48	112.63 ± 14.42	<0.001**
15 minutes	118.64 ± 15.36	102.94 ± 10.58	110.79 ± 15.32	<0.001**
20 minutes	118.81 ± 15.66	106.16 ± 10.12	112.49 ± 14.58	<0.001**
30 minutes	118.18 ± 13.95	111.24 ± 12.88	114.71 ± 13.82	0.003**
40 minutes	119.39 ± 15.17	113.39 ± 14.76	116.39 ± 15.21	0.022*
50 minutes	121.16 ± 16.41	115.78 ± 15.36	118.47 ± 16.06	0.052+
60 minutes	121.72 ± 16.16	117.85 ± 15.48	119.78 ± 15.88	0.160
70 minutes	123.53 ± 16.54	119.99 ± 15.41	121.72 ± 16.01	0.206

The baseline diastolic blood pressure values were 82.27 ± 11.69 and 81.97 ± 9.83 in Group A and Group B, respectively. There was a statistically significant difference between the two groups in diastolic blood pressure at 5 minutes, 10 minutes, 20 minutes, 30 minutes, 40 minutes, 50 minutes, 60 minutes, and 70 minutes (p-value <0.05). There was no statistically significant difference between the two groups in diastolic blood pressure at 0 minutes (p-value >0.05) (Table 6).

**Table 6:** Diastolic blood pressure comparison of the two groups

Diastolic Blood Pressure (mmHg)	Group A ( N=67) Mean ± SD	Group B ( N=67) Mean ± SD	Total ( N=134) Mean ± SD	P Value
Baseline	82.27 ± 11.69	81.97 ± 9.83	82.12 ± 10.75	0.872
0 minutes	83.63 ± 10.64	81.76 ± 10.13	82.69 ± 10.39	0.301
5 minutes	78.28 ± 13.58	65.51 ± 9.37	71.9 ± 13.27	<0.001**
10 minutes	75.88 ± 11.41	60.99 ± 8.55	68.43 ± 12.52	<0.001**
15 minutes	71.73 ± 16.41	63.63 ± 9.64	67.68 ± 14.01	<0.001**
20 minutes	71.64 ± 16.25	64.21 ± 9.17	67.93 ± 13.66	<0.001**
30 minutes	74.4 ± 12.91	67.55 ± 11.73	70.98 ± 12.76	0.002**
40 minutes	74.51 ± 12.66	68.01 ± 12.35	71.26 ± 12.88	0.003**
50 minutes	75.85 ± 13.46	69.64 ± 13.55	72.75 ± 13.81	0.009**
60 minutes	75.73 ± 12.85	70.28 ± 13.37	73.01 ± 13.35	0.018*
70 minutes	77.37 ± 13.66	70.95 ± 14.45	74.18 ± 14.37	0.012*

The baseline mean arterial blood pressure values were 95.95 ± 13.33 and 93.52 ± 8.96 in Group A and Group B, respectively. There was a statistically significant difference between the two groups in mean atrial blood pressure at 5 minutes, 10 minutes, 20 minutes, 30 minutes, 40 minutes, 50 minutes, and 60 minutes (p-value <0.05). There was no statistically significant difference between the two groups in baseline mean arterial blood pressure at 0 min (p-value >0.05) (Table 7).

**Table 7:** Mean arterial blood pressure comparison between the two groups of patients

Mean Arterial Blood Pressure (mmHg)	Group A (N=67) Mean ± SD	Group B (N=67) Mean ± SD	Total (N=134) Mean ± SD	p-value
Baseline	95.95 ± 13.33	93.52 ± 8.96	94.73 ± 11.38	0.218
0 minutes	105.87 ± 12.54	102.84 ± 11.96	104.35 ± 12.3	0.155
5 minutes	102.75 ± 21.91	90.36 ± 12.99	96.55 ± 18.99	<0.001**
10 minutes	80.67 ± 10.49	75.93 ± 9.39	78.3 ± 10.2	0.007**
15 minutes	76.91 ± 14.64	70.36 ± 9.05	73.63 ± 12.56	0.002**
20 minutes	92.66 ± 11.94	77.42 ± 8.97	85.04 ± 13	<0.001**
30 minutes	92.1 ± 12.45	81.81 ± 8.06	86.96 ± 11.66	<0.001**
40 minutes	88.79 ± 13.24	79.94 ± 8.38	84.37 ± 11.9	<0.001**
50 minutes	89.04 ± 12.65	80.75 ± 9.49	84.9 ± 11.89	<0.001**
60 minutes	88.7 ± 13.04	80.67 ± 9.32	84.69 ± 11.99	<0.001**
70 minutes	89.79 ± 13.05	84.69 ± 11.33	87.16 ± 12.41	0.018*

The visual analogue scale score at the time of the first analgesic request was 3.74 ± 1.14 in Group A and 5.81 ± 0.74 in Group B. There was a statistically significant difference between the two groups in the visual analogue scale score at 6 hours, 12 hours, and 24 hours after surgery (p-value <0.001) (Table 8).

**Table 8:** Visual analogue scale score (1–10) comparison of the two groups

Visual Analogue Scale Score (1-10)	Group A (N=67) Mean ± SD	Group B (N=67) Mean ± SD	Total (N=134) Mean ± SD	p-value
At the time of the first analgesic request	3.74 ± 1.14	5.81 ± 0.74	4.78 ± 1.41	<0.001**
6 hours after surgery	4.08 ± 1.11	5.78 ± 0.76	4.93 ± 1.27	<0.001**
12 hours after surgery	4.15 ± 0.73	5.66 ± 0.73	4.91 ± 1.05	<0.001**
24 hours after surgery	4.18 ± 0.89	5.51 ± 0.79	4.85 ± 1.07	<0.001**

## Discussion

SCSEA provides rapid onset, prolonged duration, less incidence of toxicity from local anesthetics, and postoperative analgesia. SA is a simple and quick technique, but it has a risk of severe hypotension. SCSEA can be used in patients undergoing orthopedic surgery due to hemodynamic stability. SCSEA is increasingly used as an anesthetic method for orthopedic surgeries and is considered an advanced regional anesthetic method with many advantages. The main advantage of this method is it reduces the chances of anesthesia-induced hypotension to a great extent due to the use of an initial low dosage of local anesthetic followed by an epidural top-up [10-12].

The present study included a total of 134 participants who were divided into two groups of 67 each. Group A participants were administered SCSEA, and Group B was administered SA. The mean age in both groups was around 39 years, and the majority of participants belonged to ASA Grade I in both groups.

The onset of sensory blockade in the SCSEA group was 7.15 ± 0.75 and 5.01 ± 0.88 in the SA group. This indicates that the SA group exhibited a faster onset of sensory block. The time taken to achieve motor block was 9.64 ± 1 in the SCSEA group and 7.13 ± 0.8 in the SA group. The values reflect that the SA group exhibited better motor blocking. The time for two-segment regression in the SCSEA group was 108.34 ± 29.5, while in the SA group, it was 135.24 ± 12.88. This shows that SA had a better sensory block.

In the current research, hemodynamic instability was considered when hypotension (systolic blood pressure below 90 mmHg or a 25% decrease in systolic blood pressure from the baseline value) and bradycardia with a pulse rate below 60 bpm. Hemodynamic parameters, such as the baseline pulse rate (bpm), were comparable in the two groups, which were 84.28 ± 10.38 and 84.48 ± 9.78 in SCSEA and SA, respectively. There was a statistically significant difference between the two groups in pulse rate at 5 minutes, 10 minutes, and 50 minutes (p-value <0.05).

In the SCSEA group, there was a fall in systolic blood pressure only after 10 minutes of an epidural which was 118.81 ± 15.66 and gradually increased as time proceeded until it was 123.53 ± 16.54 after 70 minutes of an epidural. In the SA group, a significant fall in systolic blood pressure was observed after 5 minutes of SA, which was 104.91 ± 10.48, and it remained on the lower side for a long time (p-value <0.05).

In the SCSEA group, there was not much fall in diastolic blood pressure; after 10 minutes of epidural, it was 71.64 ± 16.25, which remained the same throughout the procedure. However, in the SA group, there was a significant reduction in diastolic blood pressure after 5 minutes of SA. It was 60.99 ± 8.55 and remained on the lower side thereafter (p-value <0.05). This indicates that SCSEA had better hemodynamics when compared to SA. Further, the visual analogue scale score at the time of the first analgesic request in the SCSEA group was 3.74 ± 1.14, and in the SA, it was 5.81 ± 0.74. A good level of analgesia indicates greater patient comfort.

In the present study, the mean onset of sensory block in SCSEA was 7.15 ± 0.75 minutes, and in the SA group, it was 5.01 ± 0.88 minutes. This observation of the SCSEA group taking a long time for the onset of sensory block is similar to that observed in a similar study by Bhattacharya et al. In their study, the mean onset of sensory block was 10.10 ± 1.1 minutes in the SCSEA group (5 mg of 0.5% hyperbaric bupivacaine plus fentanyl 20 µgm for spinal block + epidural catheter) and 9.8 ± 1.0 minutes in the SA group. Delay in onset in the SCSEA group is due to intentionally low initial anesthetic dosage to combat hypotension [10].

The mean time (±SD) for sensory blockade in the SCSEA group was longer, i.e., 7.15 ± 0.75, while in the SA group, it was 5.01 ± 0.88. Therefore, the SA group showed a faster onset of sensory block. Begum et al. [11] observed that the mean (±SD) duration of anesthesia was significantly longer in the SCSEA group than in the SA group (256.57 ± 33.56 minutes versus 214.71 ± 18.03 minutes, P < 0.001), and the mean (±SD) time to achieve a target level of sensory block was significantly longer in the SCSEA group than in the SA group (11.21 ± 2.2 minutes versus 3.5 ± 1.5 minutes, P < 0.001) [11].

In the present study, the SCSEA group showed a reduction in systolic blood pressure only after 20 mins of an epidural, which dropped to 118.81 ± 15.66 from 124.9 ± 13.02, and in the SA group 106.16 ± 10.12 from 116.61 ± 10.98. These changes indicate a significant drop in systolic blood pressure in the SA group when compared to the SCSEA group. The study by Holmström et al. [12] noted that the median level in patients receiving epidural blocks was T8 (range T3-T12), in patients receiving spinal blocks was T8 (range T4-T10), and in patients receiving combined spinal epidural blocks was T6 (range T3-T10) (P < 0.05). No differences were noted among the groups regarding the incidence of hypotension [12].

In the present study, a considerable fall in the mean arterial blood pressure from 20 to 40 minutes was noted in the SA group, whereas it was maintained more or less constant at all times in the SCSEA group (P < 0.05). This indicates that SCSEA maintains hemodynamic stability. This observation is similar to that found in the study by Mutahar et al. [13]. A prospective, randomized, double-blind study reported a significant decrease in the mean arterial blood pressure in SCSEA in comparison with SA. From 2 minutes to 60 minutes, there was a fall in the mean arterial blood pressure in the spinal group in comparison to the SCSEA group (P < 0.05) [13].

Here, we found that two-segment regression was faster in SCSEA than in SA as reported by Yun et al. Among patients posted for lower limb surgeries, those who received only SA (10 mg of spinal bupivacaine) and SCSEA at different doses (7.5 mg of spinal bupivacaine + epidural 1.5% lidocaine 10 ml) or (5 mg of spinal bupivacaine + epidural 1.5% lidocaine 10 ml), the regression of sensory block was faster in the SCSEA group with 5 mg spinal bupivacaine than in the other two groups (P = 0.004) (Yun et al., 2014) [14].

The study proves that the onset of motor block was faster in SA (7.13 ± 0.8) than in SCSEA (9.64 ± 1), and analgesia was longer in the SCSEA group and was proven in the study by Talikota et al. [15]. In a randomized, single-blind controlled study contrasting the efficacy and safety of the SCSEA technique and SA for lower abdominal surgeries, the time taken for onset of anesthesia in the SA group was 5.48 minutes, compared to 7.40 minutes in the SCSEA group. Analgesia lasted for 115.6 minutes with the SA and 124.5 minutes in SCSEA [15].

Sundar and Mundwadkar (2017) conducted a study to compare combined spinal epidural and epidural block in the lower limb and abdominal surgeries and found that the majority of patients who received combined spinal epidural had good quality analgesia when compared to the epidural route alone. This relationship is very significant in the SCSEA group with a p-value [16].

Limitations

The study findings are based on a single center. If it can be a multi-center study, the results can be more significant. We have addressed ASA Grades 1 and 2 patients. The advantage of SCSEA with low doses of local anesthetic can be attempted on ASA Grades 3 and 4 patients with less hypotension when compared to a single shot spinal with a large volume of the drug. The current study was not conducted in our institute previously. Further research is advised in lower abdominal surgeries with various sensory levels as a result of the study being limited to orthopedic procedures. Other limitations are the involvement of different anesthetists and surgeons.

Summary

SCSEA has a substantial advantage in that it allows for the administration of low-dose intrathecal local anesthetics while knowing that the epidural catheter can be utilized to extend the block as needed. Due to fast sympathetic blocking, SA can cause a quick onset of hypotension. In patients with a low cardiac reserve or less intravascular volume, this can be dangerous. The first low anesthetic dose injected intrathecally can induce a speedy onset of a block with an SCSEA approach, but the epidural catheter inserted afterward can be used to ensure an acceptable level of the sensory blockade and to prolong the block for surgical anesthesia or postoperative analgesia. Enhanced cephalad spread of the spinal anesthetic in the intrathecal region can result from epidural bolus injection and thecal sac compression during SCSEA. The study compared the clinical effects of SCSEA and SA in patients undergoing orthopedic surgeries. In both groups, the target of the level of sensory block was the same. The study concluded that SCSEA provides more hemodynamic stability due to less intrathecal drug and slower onset of epidural top-up, and SCSEA provides a post-operative analgesic effect for a longer duration in comparison with SA.

## Conclusions

This study demonstrates that SCSEA with a titrated dose of local anesthetic has greater hemodynamical stability (mean arterial blood pressure p- value <0.05) and lesser changes in systolic and diastolic blood pressure values during different time intervals with limited use of vasopressors in intraoperative period and extended analgesic effect postoperatively, whereas SA with a larger dose of local anesthetic shows a sudden change in hemodynamics but reveals greater sensory blocks.
